# Skeletal Muscle UCHL1 Negatively Regulates Muscle Development and Recovery after Muscle Injury

**DOI:** 10.3390/ijms25137330

**Published:** 2024-07-04

**Authors:** Ryan Antony, Katherine Aby, Morgan Montgomery, Yifan Li

**Affiliations:** Division of Basic Biomedical Sciences, Sanford School of Medicine, University of South Dakota, Vermillion, SD 57069, USA; ryan.m.antony@coyotes.usd.edu (R.A.); katherine.aby@coyotes.usd.edu (K.A.); morgan.montgomery@coyotes.usd.edu (M.M.)

**Keywords:** UCHL1, skeletal muscle, ischemia-reperfusion injury, myogenesis, inflammation

## Abstract

Ubiquitin C-terminal hydrolase L1 (UCHL1) is a deubiquitinating enzyme originally found in the brain. Our previous work revealed that UCHL1 was also expressed in skeletal muscle and affected myoblast differentiation and metabolism. In this study, we further tested the role of UCHL1 in myogenesis and muscle regeneration following muscle ischemia-reperfusion (IR) injury. In the C2C12 myoblast, UCHL1 knockdown upregulated MyoD and myogenin and promoted myotube formation. The skeletal muscle-specific knockout (smKO) of UCHL1 increased muscle fiber sizes in young mice (1 to 2 months old) but not in adult mice (3 months old). In IR-injured hindlimb muscle, UCHL1 was upregulated. UCHL1 smKO ameliorated tissue damage and injury-induced inflammation. UCHL1 smKO also upregulated myogenic factors and promoted functional recovery in IR injury muscle. Moreover, UCHL1 smKO increased Akt and Pink1/Parkin activities. The overall results suggest that skeletal muscle UCHL1 is a negative factor in skeletal muscle development and recovery following IR injury and therefore is a potential therapeutic target to improve muscle regeneration and functional recovery following injuries.

## 1. Introduction

Skeletal muscle is one of the most dynamic tissues in the human body, accounting for approximately 40% of total body mass and containing 50–70% of all body proteins [1]. Not only is skeletal muscle responsible for locomotion, but it also contributes significantly to bodily functions such as metabolism, thermogenesis, and energy homeostasis [1,2,3]. Similar to all other tissues, skeletal muscle must be maintained in order to keep up with mechanical and chemical requirements; two of the major mechanisms behind skeletal muscle maintenance and repair are myogenesis and mitophagy [4,5].

The formation of skeletal muscle, commonly referred to as myogenesis, is critical during embryonic development. Additionally, myogenesis is also critical for the regeneration of adult skeletal muscle following periods of atrophy or injury, the replenishment of myogenic cells in day-to-day maintenance, and repair of skeletal muscle following injury [6]. The fate of satellite cells differentiating into muscle fibers during myogenesis depends heavily on several myogenic regulatory factors such as MyoD and myogenin which are responsible for myoblast differentiation and myotube formation, respectively [4]. The activity of these regulatory factors is crucial during myogenic repair following skeletal muscle injury [7].

Ischemia-reperfusion (IR) injury is caused by restricted blood flow for an extended period of time followed by the return of sufficient blood flow; it is the most common skeletal muscle injury and is often a result of trauma such as surgical procedures and tourniquet application [8,9,10,11,12]. Myogenesis is a critical process necessary for functional skeletal muscle recovery following IR injury [13,14,15]. Despite this, the mechanisms underlying the myogenic response following IR injury in skeletal muscle remain to be fully understood.

Ubiquitin C-terminal hydrolase L1 (UCHL1) is a deubiquitinating enzyme that was originally found in the brain [16]; however, UCHL1 has also been found to be expressed in other tissues including the pancreas, liver, spleen, cancerous tissue, and skeletal muscle [17,18,19]. Our previous studies have shown that skeletal muscle UCHL1 regulates oxidative activity, mTORC1 activity, and myogenesis [20,21,22]. In this study, we report how UCHL1 affects muscle growth both during development and regeneration, as well as how it affects mitochondrial remodeling following skeletal muscle injury.

## 2. Results

### 2.1. UCHL1 Knockdown Promotes Myotube Differentiation In Vitro

**Figure 1:**
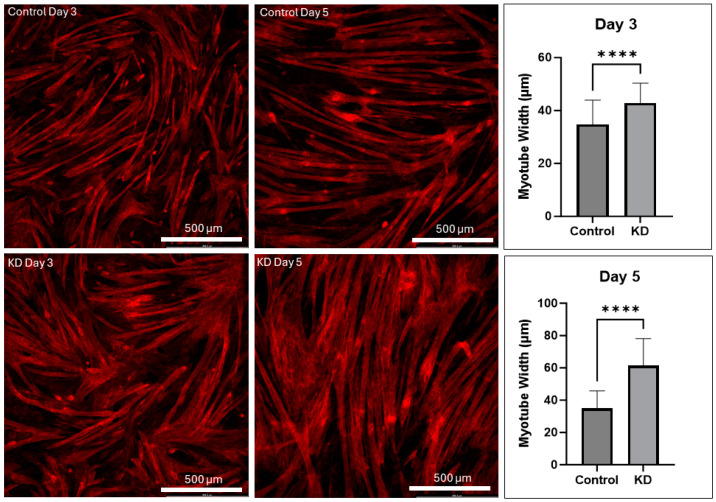
UCHL1 KD promotes myotube differentiation. Representative beta-actin immunofluorescent images and myotube width quantification between control (top) and UCHL1 knockdown (bottom) from day 3 (left) and day 5 (right), **** *p* < 0.00005, n = 60 fibers per group per time point.

UCHL1 was knocked down in C2C12 myoblasts, and cells were collected after 1, 3, and 5 days of differentiation. Myotubes were stained using Beta-Actin at each time point; the myotube number and width were significantly increased in UCHL1 KD cells when compared to control cells (Figure 1). Western blot showed that the protein expression of myogenin and MyoD was also upregulated in KD cells at each time point compared to control cells (Supplemental Figure S1); however, due to the small sample size (n = 2 dishes for each group at each time point), these data are only preliminary. These data are consistent with our previous findings that UCHL1 knockdown promotes C2C12 differentiation and myotube formation [22].

### 2.2. UCHL1 Negatively Affects Fiber Size In Vivo

**Figure 2:**
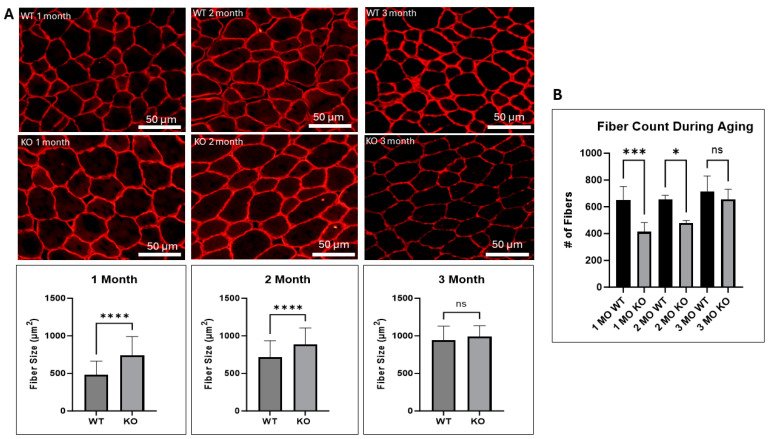
UCHL1 smKO increases fiber size and type 2b fibers. (**A**) Dystrophin immunofluorescent images and quantification between TA muscle sections from male WT (top) and UCHL1 smKO (bottom) mice at 1-month (left), 2-month (middle), and 3-month (right) time points, n = 60 fibers; (**B**) the quantification of the number of muscle fibers compared between WT and UCHL1 smKO mice at 1, 2, and 3 months of age, n = 60 fibers. * *p* < 0.05, *** *p* < 0.0005, **** *p* < 0.00005, ns = not significant.

**Figure 3:**
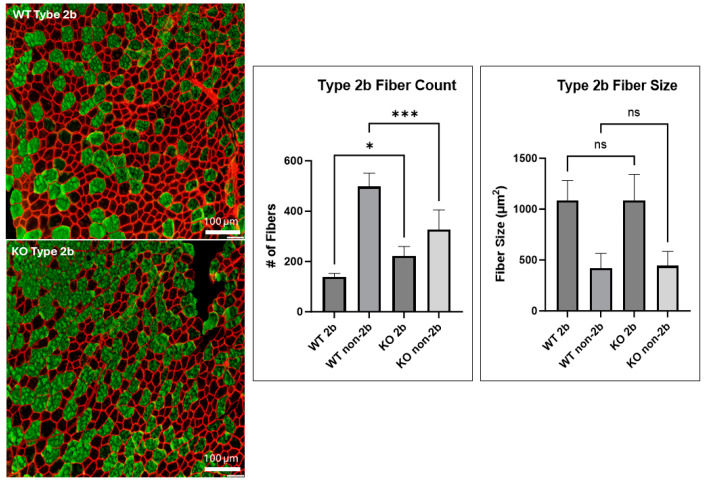
UCHL1 smKO increases type 2b muscle fibers. The immunofluorescent staining of dystrophin (red) and BFF3 (green), comparing type 2b muscle fibers between the TA muscle of 3-month-old WT and UCHL1 smKO mice (left), fiber size quantification (middle), and fiber number quantification (right). * *p* < 0.05, *** *p* < 0.0005, ns = not significant. # = number.

Given that myoD and myogenin are key regulators of myoblast differentiation, and that these proteins were upregulated as a result of UCHL1 knockdown in vitro, we assessed how UCHL1 may affect muscle growth in vivo using our UCHL1 skeletal muscle-specific knockout (smKO) mice. Compared to the flox control mice, UCHL1 smKO mice exhibited a significantly increased muscle fiber diameter at 1 and 2 months but not at 3 months (Figure 2A). Interestingly, developing muscles from UCHL1 smKO mice at 1 and 2 months of age exhibit significantly less muscle fibers than WT mice (Figure 2B). These data may suggest that UCHL1 had an inhibitory effect on muscle fiber differentiation and fusion at an early age. Moreover, staining with fiber type-specific antibodies showed that 3-month-old UCHL1 smKO mice exhibited increased type 2b muscle fibers (shown using BFF3) when compared to their WT counterparts (Figure 3) despite muscle fiber size showing no significant differences, suggesting that UCHL1 may specifically be inhibitory on glycolytic fast twitch fibers.

### 2.3. Non-Invasive Model of Hindlimb Ischemia-Reperfusion Injury

**Figure 4:**
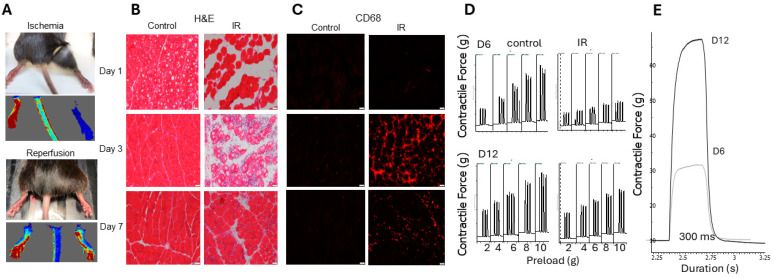
Non-invasive hindlimb ischemia-reperfusion injury model. (**A**) Live (top of set) and laser doppler (bottom of set) images during ischemia (top) and reperfusion (bottom); (**B**) H&E staining images of TA muscle sections from control limb (left) and IR limb (right) at days 1 (top), 3 (middle), and 7 (bottom), scale bars represent 20 μm; (**C**) CD68 immunofluorescent images of TA muscle sections from control limb (left) and IR limb (right) at days 1 (top), 3 (middle), and 7 (bottom), scale bars represent 20 μm; (**D**) in situ contractile force measurements from preload force tests on control limbs (left) and IR limbs (right) at day 6 (top) and day 12 (bottom); (**E**) in situ contractile force chart showing contractile force and duration of contraction between day 6 and day 12 tests from injured muscle. Preliminary data were obtained from male and female mice at 3 months of age.

To study the role of UCHL1 in muscle injury and recovery, we developed a model of non-invasive hindlimb ischemia-reperfusion (IR) injury based on a previously published study [23]. During ischemia, there was complete restriction of blood flow (Figure 4A). Using H&E and an antibody for CD68, a proinflammatory macrophage marker, tissue staining showed that IR caused severe tissue damage at days 1 and 3; the majority of proinflammatory macrophage infiltration occurs around 3 days of reperfusion, whereas the muscle itself does not exhibit visible regeneration until 7 days after reperfusion (Figure 4B,C). Further, in situ contractile testing displayed that muscle had greater functional performance at 12 days after injury when compared to muscle at 6 days after injury (Figure 4D,E).

### 2.4. Effects of UCHL1 Knockout during Injury

**Figure 5:**
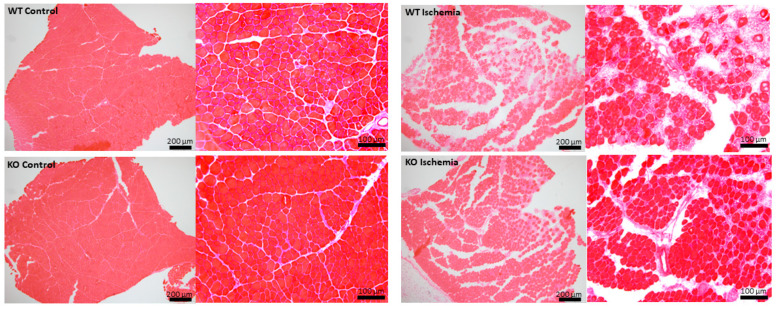
The effects of UCHL1 smKO following IR injury. H&E staining images of TA muscle sections taken from 3-month-old male control mice (top) and UCHL1 smKO mice (bottom). Both control (left) and IR (right) limbs are represented at 4× magnification (left side) and 10× magnification (right side) 7 days after injury.

To examine how UCHL1 affects muscle injury, we subjected 3-month-old flox control and UCHL1 smKO mice to hindlimb IR injury. H&E staining showed that IR-injured muscle from UCHL1 smKO mice had visibly less damaged muscle fiber bundles and less leukocyte infiltration when compared to the injured muscle of control mice (Figure 5). Given the absence of centrally located nuclei within the muscle fibers of IR sections from UCHL1 smKO mice, the data may suggest that the muscle of UCHL1 smKO mice is potentially more resistant to damage.

### 2.5. UCHL1 smKO Mice Exhibit Upregulated Myogenic Factors in Injured Muscle

**Figure 6:**
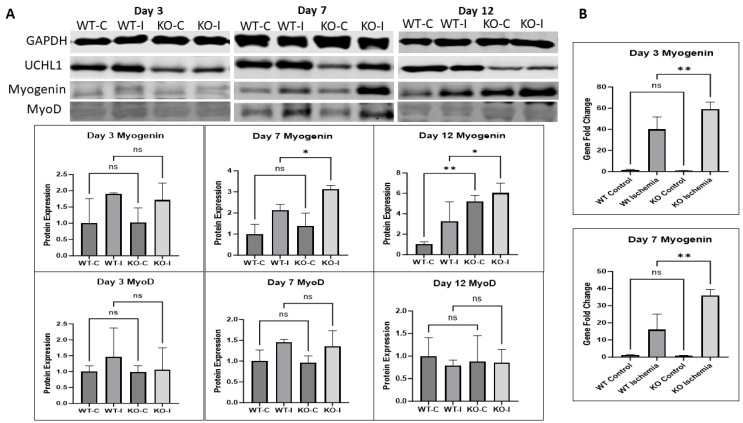
UCHL1 smKO upregulates myogenic factors after injury. (**A**): Raw images and quantifications of Western blots for GAPDH, UCHL1, myogenin, and MyoD at day 3 (left), day 7 (middle), and day 12 (right) comparing soleus from 3-month-old male WT control, WT IR, smKO control, and smKO IR limbs, n = 3 per group; (**B**): QPCR quantification of gene fold change in myogenin from day 3 (top) and day 7 (bottom) from plantaris of WT control, WT IR, smKO control, and smKO IR limbs, n = 3 per group. * *p* < 0.05, ** *p* < 0.005, ns = not significant.

To further examine the effects of UCHL1 on the regeneration of injured muscle, Western blot and qPCR were used to look at myogenic markers. The myogenin protein level in IR muscle was no different between control and UCHL1 smKO mice at day 3 but was significantly upregulated in UCHL1 smKO mice at day 7. Interestingly, the knockout mice exhibited significantly increased myogenin protein expression in both the control and injured muscle at day 12 when compared to control mice (Figure 6A). qPCR showed that myogenin mRNA expression was significantly upregulated in the injured muscle of UCHL1 smKO mice compared to control mice both at day 3 and day 7 (Figure 6B). These results confirm that UCHL1 smKO leads to an increase in myogenic activity after IR injury. The protein level of MyoD, an early-stage myogenic marker of proliferation, was no different between control and UCHL1 smKO mice across all time points, suggesting that UCHL1 may only regulate differentiation in adult skeletal muscle regeneration.

### 2.6. UCHL1 smKO Muscle Has Improved Functional Recovery Following Injury

**Figure 7:**
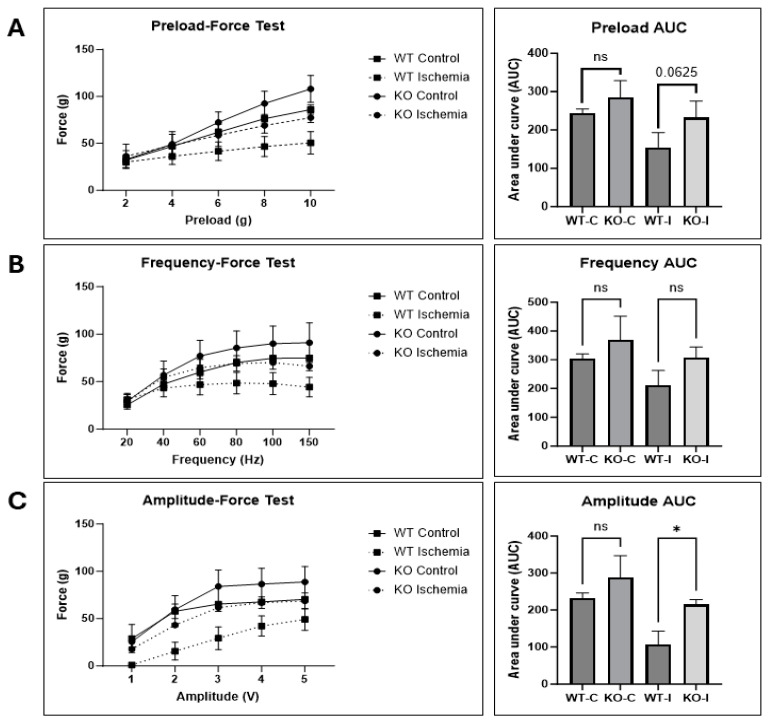
UCHL1 smKO improves functional recovery following IR injury. (**A**): Quantification of contractile force at different contraction preloads between control and injured limbs of WT and UCHL1 smKO mice 12 days after injury, and area under curve analysis; (**B**): quantification of contractile force at different contraction frequencies between control and injured limbs of WT and UCHL1 smKO mice, and area under curve analysis; (**C**): quantification of contractile force at different contraction amplitudes between control and injured limbs of WT and UCHL1 smKO mice, and area under curve analysis. * *p* < 0.05, ns = not significant. n = 3, 3-month-old male mice for each contractile test.

To test whether UCHL1 also has an effect on muscle function following skeletal muscle injury, control and UCHL1 smKO mice were subjected to in situ contractile force testing 12 days after IR injury. The injured gastrocnemius–plantaris complex in the UCHL1 smKO mice had a nearly significant increase in contractile force (Figure 7A) when subjected to several different preloads. Contractile force was not significantly different between the injured muscle of WT and UCHL1 smKO mice when subjected to various contraction frequencies (Figure 7B); however, contractile performance was significantly increased in the injured muscle of KO mice when subjected to various contraction amplitudes (Figure 7C). These data suggest that UCHL1 smKO improves muscle functional recovery after IR injury.

### 2.7. UCHL1 Regulates Mitophagy Signals

Myogenesis and muscle regeneration involve several mechanisms and signaling pathways. To assess how UCHL1 affects muscle regeneration, we measured some major signaling proteins that regulate tissue growth and found that some proteins involved in mitophagy were altered. Western blot showed Pink-1, a major regulator of mitophagy, had significantly upregulated phosphorylation in IR muscle in UCHL1 smKO mice at day 7 when compared to control mice. Parkin, the downstream protein of Pink-1, was also significantly upregulated in UCHL1 smKO IR muscle.

**Figure 8:**
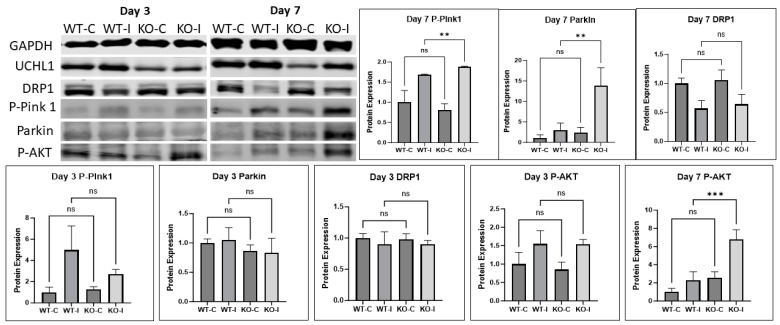
Mitochondrial remodeling is affected by UCHL1. Raw images and quantification of Western blots of GAPDH, UCHL1, DRP1, phosphor-pink1, parkin, and phosphor-AKT (S473) comparing soleus of 3-month-old male WT control, WT IR, smKO control, and smKO IR limbs from mice at day 3 and day 7, ** *p* < 0.005, *** *p* < 0.0005, ns = not significant. n = 3 mice per group.

Moreover, DRP1, the regulator of mitochondrial fission, became upregulated in UCHL1 smKO IR muscle at day 12 (Supplemental Figures S2 and S3). Additionally, AKT phosphorylation at S473, which regulates Pink-1 [24], was significantly upregulated in UCHL1 smKO mice at days 7 and 12 (Figure 8, Supplemental Figures S2 and S3). These results suggest that the promotion of myogenesis and regeneration by UCHL1 smKO may at least be in part by increasing mitophagy.

### 2.8. UCHL1 smKO Alters Inflammatory Markers

**Figure 9:**
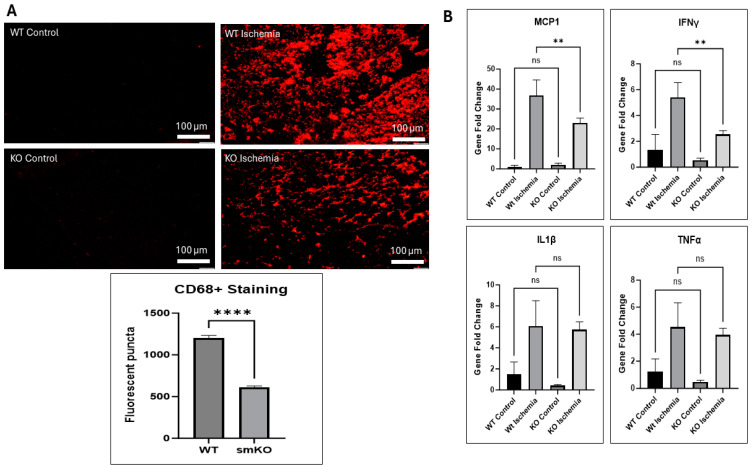
The inflammatory response alteration by UCHL1 smKO. (**A**) CD68 immunofluorescent images of TA muscle sections comparing 3-month-old male WT (top) and smKO (bottom) mice control (left) and IR (right) hindlimbs 3 days after injury; (**B**) QPCR quantification of gene fold change in MCP1 (top left), IFNγ (top right), IL1β (bottom left), and TNFα (bottom right) comparing plantaris from WT control, WT IR, smKO control, and smKO IR limbs 3 days after injury, ** *p* < 0.005, **** *p* < 0.00005, ns = not significant. n = 3 mice per group.

The inflammatory response, provided by the immune system, is essential for the maintenance of tissue homeostasis, ensuring tissue survival during infection, and healing following tissue injury [25]; however, sterile inflammation, such as with IR injury, can act as a double-edged sword by collaterally damaging healthy cells that would have otherwise been beneficial to the repair process [25,26,27]. Staining for CD68 revealed that UCHL1 smKO mice exhibited a decreased infiltration of proinflammatory macrophages after 3 days of reperfusion when compared to control mice (Figure 9A). Furthermore, qPCR displayed significantly downregulated mRNA levels of MCP1 and IFNγ in damaged muscle from UCHL1 smKO mice after 3 days of reperfusion when compared to control mice; mRNA levels of IL1β and TNFα were unchanged (Figure 9B). The data suggest that UCHL1 may also play a role in regulating the inflammatory response following skeletal muscle injury.

## 3. Discussion

UCHL1 was originally discovered as a brain-specific protein, and its role in neuronal function has been well documented [28,29]; however, despite also being found in other tissue [17,18,19], the role of UCHL1 in skeletal muscle is still relatively unknown. Previous findings from our lab have reported that UCHL1 is involved in the regulation of both skeletal muscle oxidative activity and mTORC1 signaling activity, as well as playing a role in myogenesis [20,21,22]. Consistent with our previous work, this study shows that the knockdown of UCHL1 in C2C12 myoblasts results in increased myotube width and upregulated MyoD and myogenin, the two critical myogenic factors. In vivo, this study further characterizes the role of UCHL1 in skeletal muscle fiber development in mice at various time points throughout aging. Using dystrophin immunostaining to visualize muscle fiber size, our data show that UCHL1 smKO mice have significantly increased muscle fiber size at 1 month and 2 months of age; however, muscle fiber size at 3 months showed no significant difference when compared to control mice. Additionally, muscle from 1- and 2-month-old UCHL1 smKO mice had significantly less fibers than muscle from WT mice. These results, along with UCHL1 knockdown enhancement in C2C12 differentiation in vitro, suggest that UCHL1 is a negative regulator of differentiation during myogenesis but may not be critical for adult muscle mass maintenance. However, UCHL1 smKO leads to an increase in type 2b glycolytic fast twitch fibers, suggesting that UCHL1 may also regulate muscle metabolism and thus muscle function.

Skeletal muscle has a great risk of injury, as well as a great capacity of myogenesis and regeneration after injury. In the hindlimb IR injury mouse model, the muscle is significantly damaged 1 day after injury, the majority of inflammation and neutrophil infiltration occurs around day 3, and the muscle itself appears to be morphologically healed by day 7. The data further showed that injured muscle is not functionally healed until around day 12 of reperfusion. Given the results, we chose days 3, 7, and 12 as time points to assess differences. Using this model, this study reveals for the first time that UCHL1 smKO upregulates myogenic factor myogenin, promotes muscle regeneration, and enhances functional recovery following skeletal muscle injury. Overall, these results further suggest that UCHL1 is not only a negative regulator in normal muscle development and growth but also elicits inhibitory effects in post-injury regeneration and therefore can be a potential therapeutic target for muscle injury and regeneration.

Skeletal muscle myogenesis and regeneration are regulated by multiple mechanisms and signaling pathways. The PI3K/AKT signaling pathway activity is critical for the regulation of skeletal muscle myogenesis [30,31,32]. Our Western blot results show that the phosphorylation of AKT (S473) is significantly upregulated in injured muscle from UCHL1 smKO at days 7 and 12 when compared to control mice. Our previous work has shown that UCHL1 regulates mTORC1 activity [21]; combined with the UCHL1 knockout-affected phosphorylation of AKT (S473), the results suggest that UCHL1 may regulate myogenesis via a signaling target upstream of the mTOR complexes. It is known that AKT activity is greater in fast twitch muscle; the increased phosphorylation of AKT in UCHL1 smKO muscle is also consistent with the fast twitch fiber shift seen in UCHL1 smKO muscle.

Macroautophagy, the degradation of dysfunctional and unnecessary cellular components, is critical for the reprogramming of muscle satellite cells upon leaving a quiescent state [33,34] and for cellular fusion during later stages of differentiation [35]. As a specific form of autophagy, mitophagy is also critical for skeletal muscle myogenesis and regeneration [36,37]. Damaged and depolarized mitochondria are selectively eliminated via the autophagy–lysosome system, also known as mitophagy. It has been found that enhanced mitophagy improves both mitochondrial health, as well as skeletal muscle function [5]. Our data showed that phospho-pink1 and parkin, two key regulators of mitophagy, have upregulated protein expression in UCHL1 smKO mice at day 7. The data showed a further upregulated protein expression of parkin at day 12, as well as significantly upregulated DRP1 expression, a protein essential for mitochondrial fission. These results suggest that UCHL1 regulates mitochondrial remodeling, and the UCHL1 smKO promotion of myogenesis may at least in part be due to the enhanced mitophagic response.

Inflammation has complex roles in muscle injury and regeneration. In general, inflammation caused by muscle damage is critical for containing damage, cleaning dead cells and tissue debris, and activating myogenesis. However, chronic and unresolved inflammation is detrimental for myogenesis and regeneration [38,39]. Our data showed that UCHL1 smKO mice had better morphological muscle composition 7 days after injury, as well as decreased neutrophil infiltration when compared to control mice. Furthermore, UCHL1 smKO mice had decreased proinflammatory macrophage infiltration and significantly downregulated MCP1 and IFNγ RNA expression 3 days after injury. This anti-inflammatory effect of UCHL1 smKO may also contribute to the improved regeneration; however, given the increase in glycolytic fibers in UCHL1 smKO muscle, it is also possible that the reliance on anaerobic metabolism increases resistance to hypoxic damage and reduces overall inflammation.

Overall, this study further characterizes the involvement of UCHL1 in myogenesis during skeletal muscle development, as well as providing evidence that UCHL1 regulates myogenesis following skeletal muscle injury, possibly by the regulation of the mTOR/AKT signaling pathway. This study also provides novel evidence that UCHL1 regulates mitochondrial remodeling, at least partially via the pink-1/parkin pathway. However, this study was not able to identify the direct substrates that are responsible for the improved myogenic differentiation and regeneration observed in UCHL1 smKO mice. Furthermore, the presumable resistance to muscle damage supported by decreased inflammatory markers and better morphology poses the question as to where UCHL1 becomes involved. In addition, while the upregulation of pink-1 and parkin by UCHL1 smKO strongly suggests enhanced mitophagy, more mitophagy markers should be further examined. Lastly, given that (macro)autophagy plays a role in myogenic functions, it would be beneficial to analyze autophagic flux during development and regeneration. Further studies are needed to address these limitations of this study.

## 4. Methods and Materials

### 4.1. Cell Culture

C2C12 mouse myoblasts were cultured in complete medium (CM) which consists of DMEM containing 10% FBS, 1% penicillin/streptomycin (P/S), and 1% HEPES solution until 90% confluency. Cells were then transfected in DMEM and lipofectamine RNAMAX using UCHL1 siRNA to induce the knockdown of UCHL1 with control cells being transfected with control/WT siRNA. Media were then changed to differentiating medium (DM) which consists of DMEM containing 2% horse serum, 1% P/S, and 1% HEPES solution, allowing cells to differentiate for 1–5 days before being collected and lysed for Western blot. Hydrogen peroxide treatment was conducted at a concentration of 500 μM for 12 h in DM. Myotube width was measured at the widest point of the cell.

### 4.2. Animals

The animal use in this study was approved by the University of South Dakota IACUC (protocol number 01-05-22-25D). Male mice at 1, 2, and 3 months of age were used for the developmental experiments in this study, and 3-month-old male mice were used for IR experiments. To achieve the skeletal muscle knockout (smKO) of UCHL1, breeder mice with floxed UCHL1 were crossed with mice expressing skeletal muscle-specific Cre (Myl1^tm1(cre)Sjb^/J, Jackson Labs, Bar Harbor, ME, USA) as described in our previous work [20,21] and validated in our previous publication [20]. Floxed UCHL1 mice without Cre served as controls for this study’s experiments. All above mouse strains were in C57BL/6J background. Both male and female mice were used to validate the IR model (Figure 4), but only male mice were used in the remainder of the experiments.

#### 4.2.1. Non-Invasive Hindlimb Ischemia-Reperfusion Model

Adapted from the published method [38] with modification, three-month-old male and female UCHL1 smKO and control mice were anesthetized using isoflurane inhalant (2–3%). Buprenorphine SR was administered at a concentration of 1 mg/kg to control pain after waking. An orthodontic rubber band (ORB) was placed at hip level on the right hindlimb using a McGivney ligator applicator, leaving the left hindlimb as the contralateral control. The rubber band was left in place for 90 min; complete ischemia was confirmed using laser Doppler imaging (Moor Instruments). Following the period of ischemia, the ORB was removed, allowing for reperfusion for 3–12 days. Animals were sacrificed, and tissue was collected from the IR-injured and contralateral control limbs.

#### 4.2.2. In Situ Muscle Contraction

In situ muscle contraction was also conducted as previously described [40] with modification. In brief, mice were anesthetized using a mixture of Urethane (2 mg/kg) and α-chloralose (50 mg/kg) via intraperitoneal injection. The skin of the right hindlimb was removed. The gastrocnemius–plantaris muscle complex was isolated and connected to a force transducer, and a pair of electrodes were placed at the proximal and distal ends of the gastrocnemius–plantaris complex. The muscle complex contractile function was tested with several conditions including various preload, frequency, and voltage settings. The procedure was then repeated for the contralateral hindlimb which serves as the control for this experiment. The Grass S88 stimulator was used. For all tests, the train rate was set to 1 train per second (TPS) and train duration set to 300 ms. For preload force testing (i.e., contractile force at various tension prior to stimulation), stimuli were set to 2 ms, 10 volts, and 100 Hz, and preload was changed from 2 g, 4 g, 6 g, 8 g, and 10 g. Frequency –force testing similarly used 2 ms and 10-volt settings. Preload was set to 6 g, and frequency ranged from 20, 40, 60, 80, 100, and 150 Hz. Lastly, amplitude–force testing again used 6 g preload, 2 ms duration with 100 Hz frequency. Voltage for this test utilized 1-, 2-, 3-, 4-, and 5-volt settings throughout the testing.

### 4.3. Tissue Collection

Mice were anesthetized using Urethane alpha chloralose via intraperitoneal injection as described above prior to euthanasia. Soleus and extensor digitorum longus (EDL) were collected from each hindlimb for Western blot. Tibialis anterior (TA) muscles were coated in OCT and snap-frozen in dry prechilled 2-methylbutane. The frozen TA muscles were then prepared in blocks of OCT for cryo-sectioning and finally histology/immunostaining. Lastly, the plantaris muscle from each hindlimb was collected for PCR. In non-IR experiments, future studies may use the contralateral TA muscles for Western blot and PCR assays to make the data more comparable.

### 4.4. Hematoxylin and Eosin (H&E) Staining

The H&E protocol used in this study is based off of the published H&E protocol [41]. Frozen slides/sections were taken from −80-degree storage and directly immersed into Meyers hematoxylin for 10 min at room temperature. Slides were then placed under running tap water until the water was clear. Following this, slides were then immersed in 1% Eosin at room temperature for 3 min, then rinsed under tap water until clear. Lastly, slides were dehydrated with 70% ethanol for 20 s, 95% for 20 s, and 100% ethanol for 1 min, followed by clearing in xylene for 3 min prior to mounting. The slides were examined using a Nikon microscope(Nikon Instruments, Melville, NY, USA) equipped with an Olympus DP73 camera (Olympus Life Science, Waltham, MA, USA).

### 4.5. Tissue Immunostaining

Frozen slides/sections were incubated in PBST at room temperature for 10 min prior to primary antibody incubation. Primary antibodies (Dystrophin, AbCAM, Waltham, MA, USA) were diluted to 1:100 in PBS containing 5% BSA; slides were incubated overnight in 4 º Celsius. Following incubation, slides were washed in PBS 3 times for 5 min each, then incubated with secondary antibody (1:500 dilution in PBS) for 1 h at room temperature. Slides were then washed again 3 times in PBS for 5 min each, then mounted using Fluoromount mounting solution. The slides were examined using a fluorescent Nikon microscope equipped with an Olympus DP73 camera. Using ImageJ (version 1.54), the outline of the muscle fibers measured and cross-sectional area were calculated.

### 4.6. Quantitative PCR (qPCR)

RNA was extracted from plantaris muscle tissue utilizing the Direct-zol RNA kit from Zymo research (Zymo Research, Irvine, CA, USA, Catalog #R2060-R2063) following the manufacturer’s microprep protocol. RNA samples were quantified using a nanodrop spectrophotometer (Thermo nanodrop 2000, ThermoFisher Scientific, Waltham, MA, USA), and the concentrations were normalized. A total of 10 μL of normalized sample was then mixed with an equal amount of cDNA reverse transcriptase master mix (Thermo Catalog #4368814, ThermoFisher Scientific, Waltham, MA, USA) and subjected to a reverse transcription reaction.

A total of 1 μL of cDNA was then mixed with 10 μL of PowerUp SYBR green master mix (Catalog #A25742, ThermoFisher Scientific, Waltham, MA, USA), 2 μL of RNase free water, and 2 μL of primer (IDT 18 s, IDT myogenin). Sample mixtures were then subjected to thermal cycling using the Applied Biosystems Step One Plus (Applied Biosystems, Waltham, MA, USA).

The primers used in this study were purchased from Integrated DNA Technologies (IDT): MCP1 (Forward AGG TGT CCC AAA GAA GCT GTA; Reverse: ATG TCT GGA CCC ATT CCT TCT), IFNγ (IDT predesigned qPCR primer, Assay ID: Mm.PT.58.41769240), IL1β (IDT predesigned qPCR primer, Assay ID: Mm.PT.58.41616450), and TNFα (IDT predesigned qPCR primers, Assay ID: Mm.PT.58.29509614).

### 4.7. Western Blot

Western blot was conducted as described in our previous publications [20,42,43]. Briefly, cells, or soleus and EDL muscle samples were homogenized, the protein concentrations were determined using a BCA assay, and the equal amount of total protein was mixed with loading buffer and heated at 90 °C for 10 min. Protein samples were subjected to SDS-PAGE electrophoresis and blotted with primary and secondary antibodies, then visualized by a LICOR scanner. In order of appearance, the antibodies used in this study are as follows: Beta-Actin (Santa Cruz Biotechnologies 47778, Dallas, TX, USA), UCHL1 (Protein Tech 14730-1-AP, Rosemont, IL, USA), Myogenin (Santa Cruz 52903), MyoD (Santa Cruz 377460), GAPDH (Santa Cruz 166574), DRP1 (Cell Signaling technology 8570S, Danvers, MA, USA), P-Pink1 (Cell Signaling 89010S), Parkin (Biolegend 808501, San Diego, CA, USA), and P-AKT (Cell Signaling 4051S).

### 4.8. Data Analysis

Data are presented as the mean ± standard deviation (SD). A one-way ANOVA followed by Tukey’s post hoc test or Student’s t test was applied using GraphPad Prism software (version 10.1.2) when multiple time points were not applicable. Where multiple time points were present, a two-way ANOVA followed by Sidak’s multiple comparison tests was used. Differences were considered statistically significant at *p* < 0.05. Significant differences were denoted by * = *p* < 0.05, ** = *p* < 0.005, *** = *p* < 0.0005, **** = *p* < 0.00005.

## Data Availability

Data is contained within the article and Supplementary Materials.

## Supplementary Materials

The following supporting information can be downloaded at https://www.mdpi.com/article/10.3390/ijms25137330/s1.
