# Supplementary figures and images for: Skeletal Muscle UCHL1 Negatively Regulates Muscle Development and Recovery after Muscle Injury
Source: Int J Mol Sci. 2024 Jul 4;25(13):7330. doi: 10.3390/ijms25137330 (PMC11242864; doi:10.3390/ijms25137330)

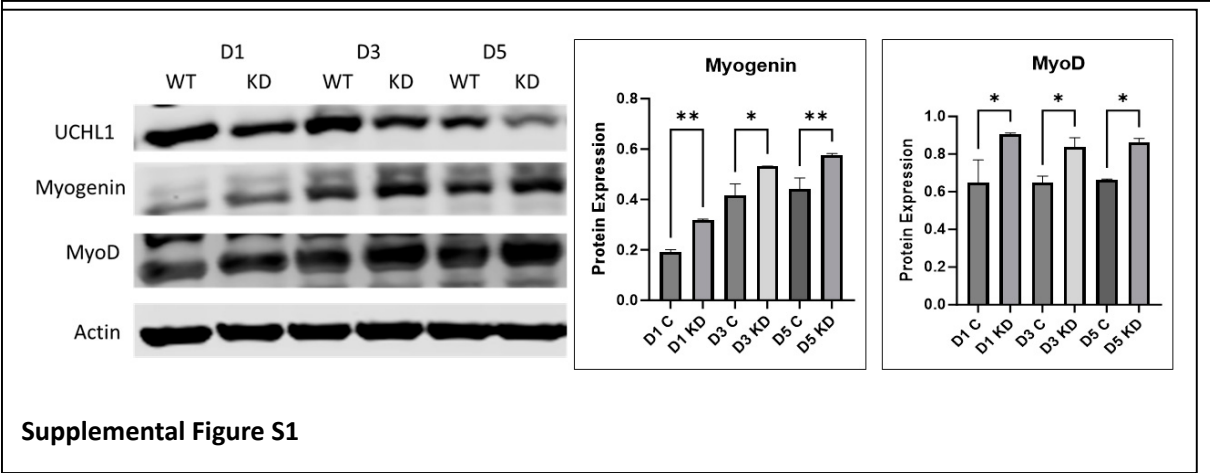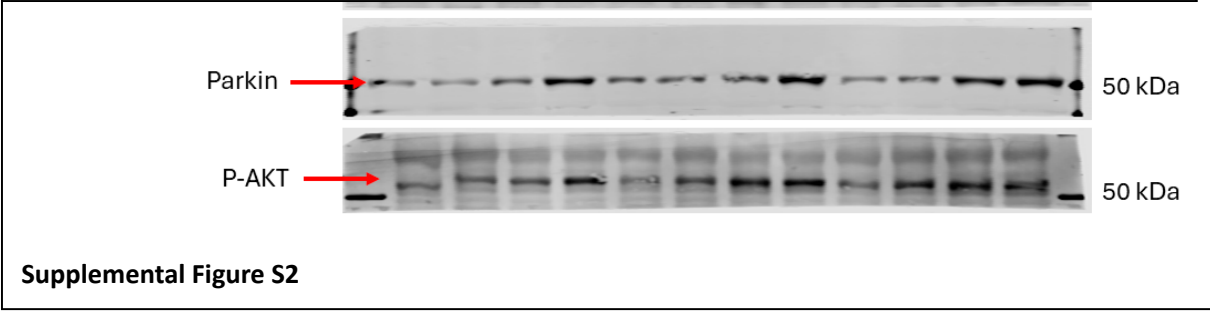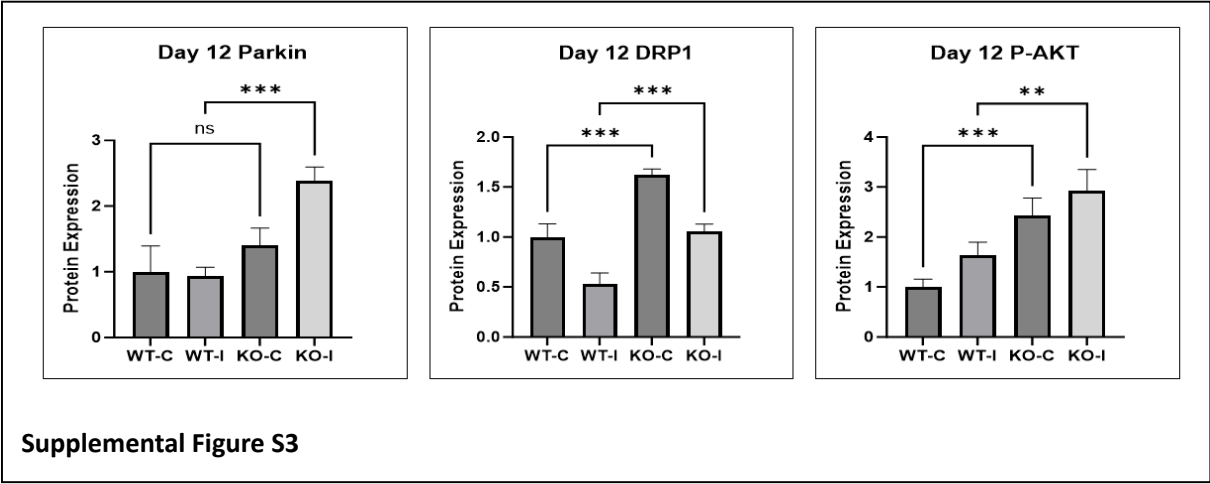

Supplement: Supplementary file 1 [file ijms-25-07330-s001.zip › ijms-3043658-supplementary.pdf]
